# Supplementary material for: Genome-wide signatures of synergistic epistasis during parallel adaptation in a Baltic Sea copepod
Source: Nat Commun. 2022 Jul 12;13:4024. doi: 10.1038/s41467-022-31622-8 (PMC9276764; doi:10.1038/s41467-022-31622-8)
Supplement: Supplementary file 1 — Supplementary Information [file 41467_2022_31622_MOESM1_ESM.pdf]

Supplementary Information

Genome-wide signatures of synergistic epistasis during parallel adaptation in a Baltic Sea copepod

Supplementary Tables

Supplementary Table 1. Parameter values used in simulations of selection with various models of epistasis. Effect sizes, starting frequencies, and effective population sizes were drawn from estimates from our empirical data. For the Positive and Negative Epistasis models, an α value of 8 was used as a starting value for the Approximate Bayesian Computation procedure, which found that an estimate of α=36.5 was consistent with our empirical data.

Fitness Function	Parameter	Default Value Hap Blocks	Default Value SNPs
Population Genetic Framework			
Multiplicative $\prod_{i=1}^{NLoci}(1+h_i s_i)$	—	—	—
Positive Epistasis $\prod_{i=1}^{NLoci}(1+h_i s_i)\cdot e^{a(x-\delta)^2}$	a	8	8
Negative Epistasis $\prod_{i=1}^{NLoci}(1+h_i s_i)\cdot e^{a(x-\delta)^2}$	a	-8	-8
Quantitative Genetic Framework			
Shifted Optimum Epistasis $e^{-\frac{((x-\delta)-\mu)^2}{\sigma^2}}$	μ<br>σ	0.4<br>0.07	0.435<br>0.0175
Directional Epistasis $(1+se^{r((x-\delta)+b)})^{-1/s}$	s<br>r<br>b	0.1<br>-15<br>0.3	0.1<br>-75<br>-0.41
Truncating Epistasis $\max\{0, 1-e^{-a((x-\delta)+b)}\}$	a<br>b	10<br>-0.25	40<br>-0.395

The Phenotype: $x = \frac{\sum_{i=1}^{NLoci} h_i s_i}{\sum_{i=1}^{NLoci} s_i}$

Horizontal Shift: $\delta = \overline{phenotype_{simulation}} - \overline{phenotype_{baseline}}$

$$h_i = \begin{cases} 0 & \text{if the individual carries 0 copies of the allele at loci } i \\ \frac{1}{2} & \text{if the individual carries 1 copy of the allele at loci } i \\ 1 & \text{if the individual carries 2 copies of the allele at loci } i \end{cases}$$

Supplementary Figures

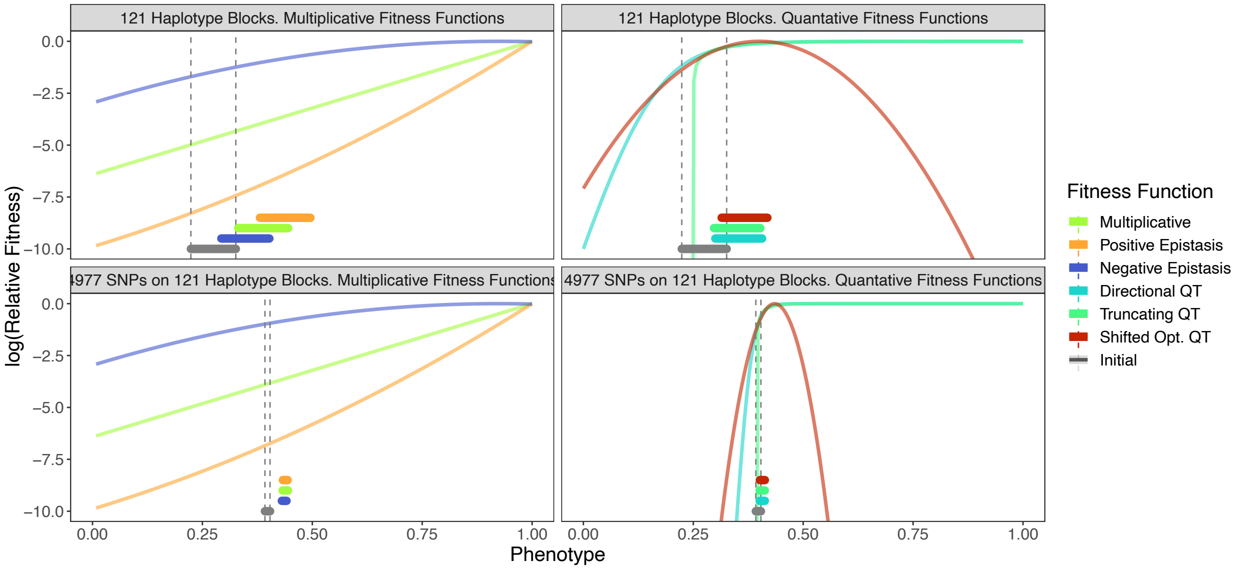

Supplementary Figure 1. Fitness functions of the models used to simulate selection in this study. Left – population genetic (multiplicative fitness) models. Right – quantitative trait models. Top – simulations of 121 independent alleles. Bottom – simulation of 4977 SNPs linked on 121 haplotype blocks. Colored horizontal bars represent the starting (grey) and ending phenotype range (different colors for each model) of the population after 10 generations of selection.

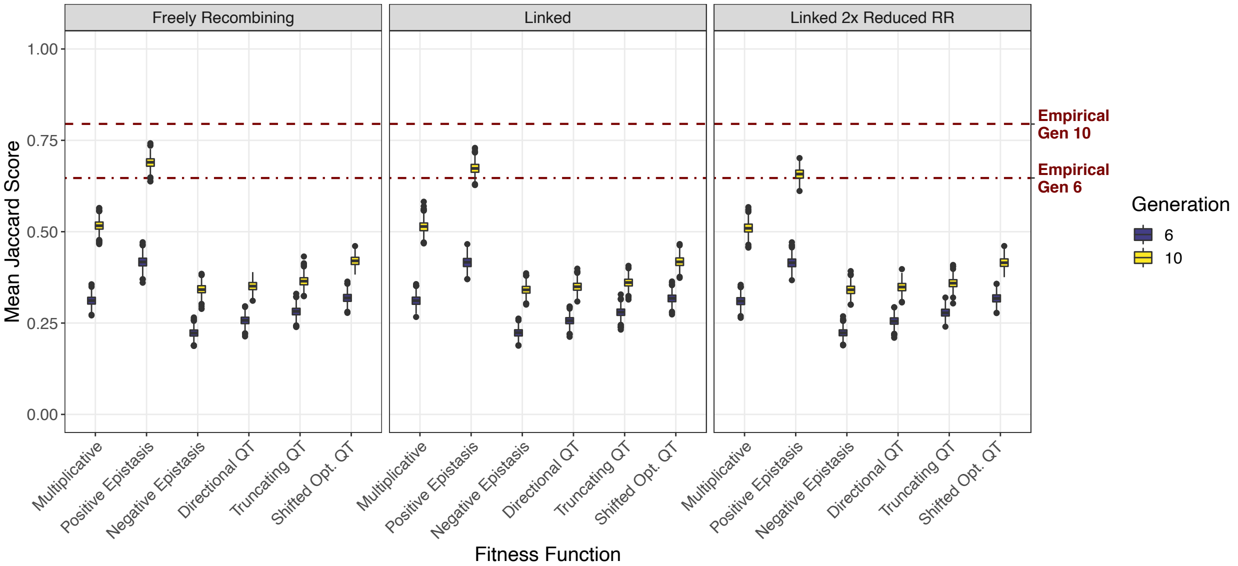

Supplementary Figure 2. The effect of recombination rate on parallel evolution in simulated data. Box plots display median (middle line), 25th and 75th percentile (box), 5th and 95th percentile (vertical line), and data beyond the 5th and 95th percentile (single points) of mean Jaccard indices between populations from 1000 simulation iterations under different models of adaptive architecture (Table 2) varying the recombination rate. Left – alleles were in linkage equilibrium. Middle – alleles recombined according to the empirical genomic distances and recombination rate of the copepod *Tigriopus californicus*. Right – same as middle, but with 50% the recombination rate, meant to exaggerate the potential effects of physical linkage. Empirical Jaccard values are plotted in dotted lines. On average across models, physical linkage with a 2x decreased recombination rate decreased the Jaccard index by 0.005 compared with freely recombining loci. Parameter values used for simulations are found in Supplementary Table 1. The positive and negative epistasis models used an α value of 8 and -8, respectively.

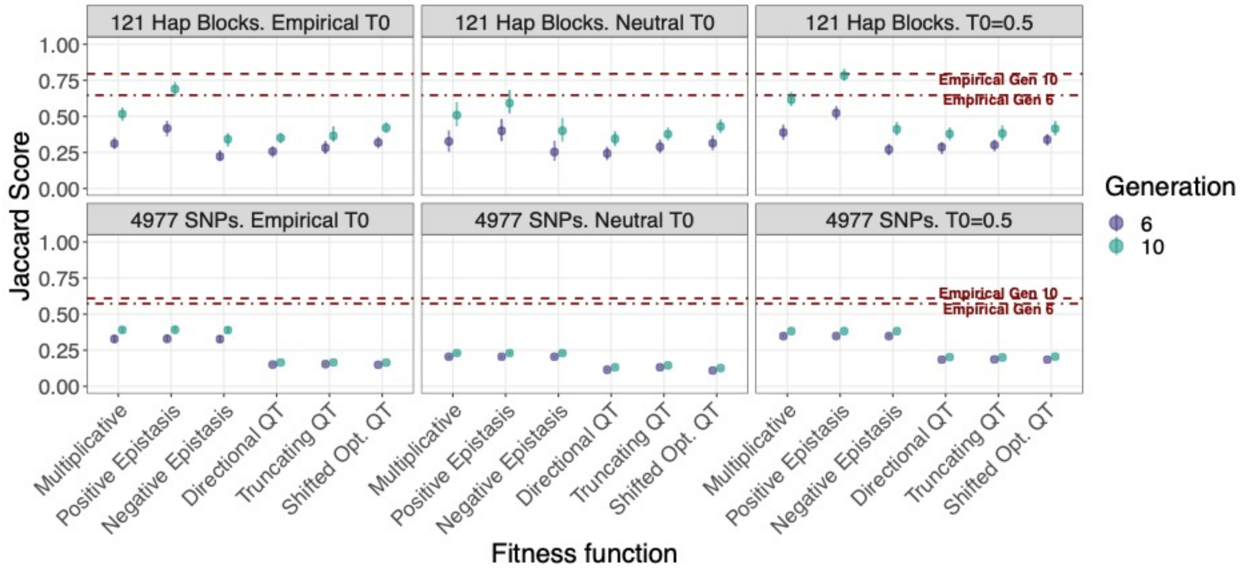

Supplementary Figure 3. The effect of starting frequency on parallel evolution in simulated data. Box plots display median (middle line), 25th and 75th percentile (box), 5th and 95th percentile (vertical line), and data beyond the 5th and 95th percentile (single points) of mean Jaccard indices between populations from 1000 simulation iterations under different models of adaptive architecture (Table 2), varying the starting allele frequency. Simulations with all alleles starting from 0.5 frequency (right) generated higher levels of parallelism than simulations using our empirical starting frequencies (left) or neutral starting frequencies (middle). Nevertheless, varying the starting frequency had a small overall effect on the Jaccard Index and did not produce levels of parallelism that matched our real data using the multiplicate or quantitative trait models. Parameter values used for simulations are found in Supplementary Table 1. The positive and negative epistasis models used an α value of 8 and -8, respectively.

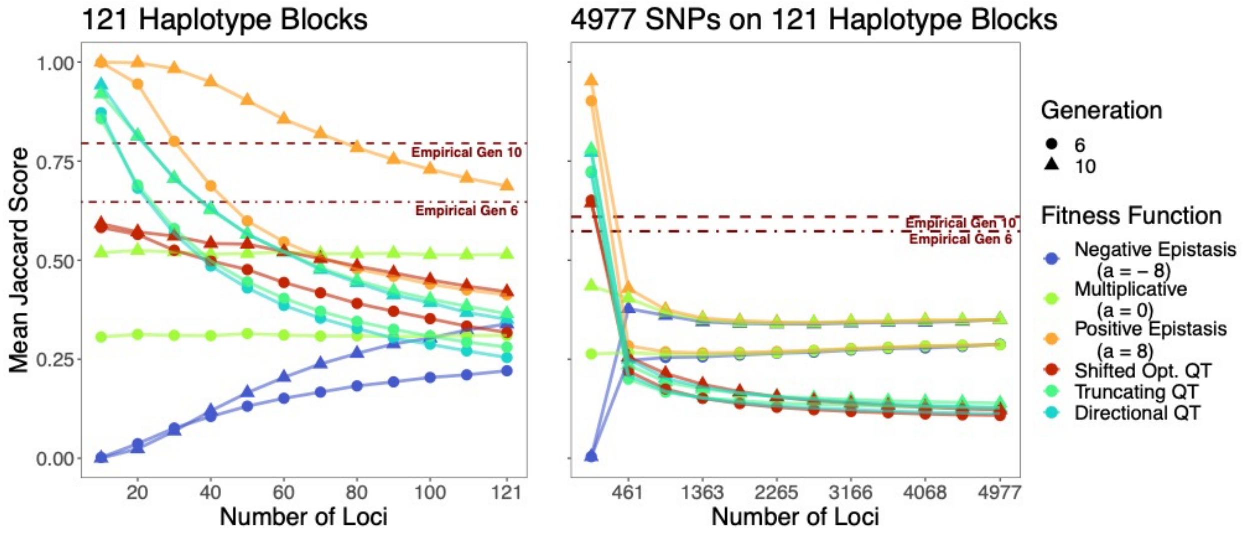

Supplementary Figure 4. The effect of varying the number of loci underlying an adaptive response on the degree of parallelism. Mean Jaccard indices (points) across 100 simulation iterations as a function of the number of loci contributing to adaptation in simulated data. Left – simulations of 121 independent alleles. Right – simulations of 4977 SNPs linked on 121 haplotype blocks. The mean Jaccard index for the empirical data is plotted in the dotted lines. The different models are described in Table 2. Parameter values used for simulations are found in Supplementary Table 1.

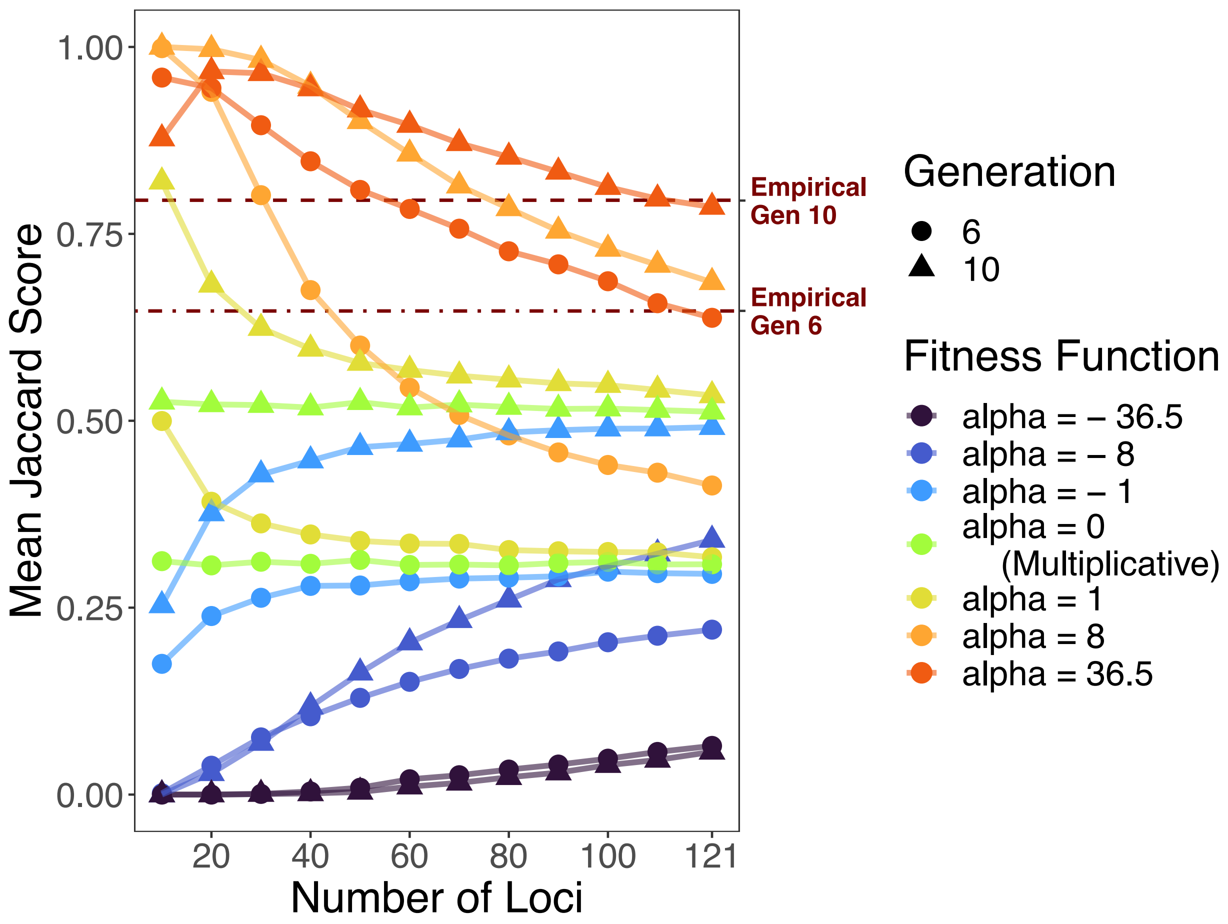

Supplementary Figure 5. The effect of varying the α parameter and number of contributing loci of the population genetic epistasis model on parallelism. Mean Jaccard indices (points) from 100 simulation iterations under each α value. The mean Jaccard index for the empirical data is plotted in the dotted lines. An α parameter of 0 is equivalent to the "multiplicative fitness" model (green). The α parameter estimated from Approximate Bayesian Computation (36.5) is plotted in dark orange. Negative values for the α parameter generate negative epistasis while positive values generate positive epistasis. Parameter values used for simulations are found in Supplementary Table 1.
