# Supplementary material for: Genome-wide signatures of synergistic epistasis during parallel adaptation in a Baltic Sea copepod
Source: Nat Commun. 2022 Jul 12;13:4024. doi: 10.1038/s41467-022-31622-8 (PMC9276764; doi:10.1038/s41467-022-31622-8)
Supplement: Supplementary file 3 — Description of Additional Supplementary Files [file 41467_2022_31622_MOESM3_ESM.pdf]

Description of Additional Supplementary Files

File Name: Supplementary Data 1
Description: Characteristics of the 121 selected haplotype blocks. Genes refers to all overlapping gene models and their approximate annotations: ID/Gene model/Uniref protein/Description

File Name: Supplementary Data 2
Description: Significantly enriched gene ontology (GO) terms for SNPs underlying selected haplotype blocks. Analysis performed using Gowinda (Kofler, R. & Schlötterer 2012). Rows highlighted in green indicate GO terms associated with iontransport and osmoregulation. p-value indicates the one-sided pvalue calculated using the empirical null distribution from 10,000 simulations. FDR-adjusted p-value used the empirical FDR correction for multiple testing as described in 'Elements of Statistical Learning' (2009): Trevor Hastie, Robert Tibshirani and Jerome Friedman, 2nd edition, pp687-690, (http://wwwstat.stanford.edu/~tibs/ElemStatLearn/)

File Name: Supplementary Data 3
Description: Collection and DNA sequencing statistics for wild samples collected for this study.

File Name: Supplementary Data 4
Description: Results of twosided Kolmogorov-Smirnov tests comparing minor allele frequency distributions for experiment-selected and non-selected SNPs. Tests were performed using the stats R package.

File Name: Supplementary Data 5
Description: Significant tests for gene flow using the f4 statistic (Reich et al. 2009). Populations refers to the four populations being tested, with labels from Supplementary Data 3. Statistical support of each of the three possible binaries trees for each combination of four populations is shown. Only significant tests (i.e. all three groupings have a |Z-score| > 2) are shown.

File Name: Supplementary Data 6
Description: Collection and sequencing statistics for the laboratory experiment samples.

File Name: Supplementary Data 7
Description: Assembly statistics for the reference transcriptome and pseudo-reference genome.

File Name: Supplementary Data 8
Description: Effective population size estimates using different methods. Estimates were made using WFABC (Foll et al. 2015) and the R package poolSeq (Taus et al. 2017). Plan II refers to the censussize-independent method of Taus et al. 2017. Plan I refers to the censussize-dependent method Taus et al. 2017. All estimates were made in 1000 SNP windows along the genome and the median reported.
