# Supplementary material for: Genome-wide signatures of synergistic epistasis during parallel adaptation in a Baltic Sea copepod
Source: Nat Commun. 2022 Jul 12;13:4024. doi: 10.1038/s41467-022-31622-8 (PMC9276764; doi:10.1038/s41467-022-31622-8)
Supplement: Supplementary file 5 — Reporting Summary [file 41467_2022_31622_MOESM5_ESM.pdf]

nature research

Corresponding author(s): David B. Stern

Last updated by author(s): June 15, 2022

Reporting Summary

Nature Research wishes to improve the reproducibility of the work that we publish. This form provides structure for consistency and transparency in reporting. For further information on Nature Research policies, see our Editorial Policies and the Editorial Policy Checklist.

Statistics

For all statistical analyses, confirm that the following items are present in the figure legend, table legend, main text, or Methods section.

n/a	Confirmed	
☐	☒	The exact sample size (*n*) for each experimental group/condition, given as a discrete number and unit of measurement
☐	☒	A statement on whether measurements were taken from distinct samples or whether the same sample was measured repeatedly
☐	☒	The statistical test(s) used AND whether they are one- or two-sided<br>*Only common tests should be described solely by name; describe more complex techniques in the Methods section.*
☐	☒	A description of all covariates tested
☐	☒	A description of any assumptions or corrections, such as tests of normality and adjustment for multiple comparisons
☐	☒	A full description of the statistical parameters including central tendency (e.g. means) or other basic estimates (e.g. regression coefficient) AND variation (e.g. standard deviation) or associated estimates of uncertainty (e.g. confidence intervals)
☐	☒	For null hypothesis testing, the test statistic (e.g. *F*, *t*, *r*) with confidence intervals, effect sizes, degrees of freedom and *P* value noted<br>*Give P values as exact values whenever suitable.*
☒	☐	For Bayesian analysis, information on the choice of priors and Markov chain Monte Carlo settings
☒	☐	For hierarchical and complex designs, identification of the appropriate level for tests and full reporting of outcomes
☐	☒	Estimates of effect sizes (e.g. Cohen's *d*, Pearson's *r*), indicating how they were calculated

Our web collection on statistics for biologists contains articles on many of the points above.

Software and code

Policy information about availability of computer code

Data collection	bcl2fastq2 v2.20, HiSeq Software Suite v3.4.0, NovaSeq Control Software v1.6
Data analysis	BLAST+ 2.7.1, BWA-MEM v0.7.17, CD-HIT v4.7, PoPoolation2, Samtools v1.3.1, Trinity v2.6.6, VarScan v2.4.3, lme4 v1.1.21, poolfstat v1.1.1, qvalue v2.14.1, ACER v1.0.2, haplovalidate v0.1.4, BBTools v38.37, Bowtie v2.3.5, Gowinda v1.12, HMMER v3.2.1, SLiM v3.7, RSEM v1.3.1, Transdecoder v5.5, Trimmomatic v0.39, TreeMix v1.13, https://github.com/jjberg2/PolygenicAdaptationCode, wtdbg v2.5, Racon v1.4.3, LiftOff v1.6.1, https://github.com/TheDBStern/Baltic_Lab_Wild, poolSeq v0.3.5, WFABC v1.1, EasyABC v1.5

For manuscripts utilizing custom algorithms or software that are central to the research but not yet described in published literature, software must be made available to editors and reviewers. We strongly encourage code deposition in a community repository (e.g. GitHub). See the Nature Research guidelines for submitting code & software for further information.

Data

Policy information about availability of data

All manuscripts must include a data availability statement. This statement should provide the following information, where applicable:

- Accession codes, unique identifiers, or web links for publicly available datasets
- A list of figures that have associated raw data
- A description of any restrictions on data availability

The raw Pool-seq data generated in this study have been deposited to the NCBI Short Read Archive under BioProject ID PRJNA844002[https://www.ncbi.nlm.nih.gov/bioproject/?term=PRJNA844002]. The E. affinis draft genome is available on Dryad[https://doi.org/10.5061/dryad.r7sqv9sdz]. Study information has been deposited to BCO-DMO[https://www.bco-dmo.org/project/816918]. Allele frequency data (SNP and haplotype block) are available on https://github.com/TheDBStern/Baltic_Lab_Wild (v.0.0.1). Data used in this study from publicly available databases include the International Council for the Exploration of the Sea (ICES) database[https://ocean.ices.dk/Helcom/Helcom.aspx?Mode=1], NCBI RefSeq[https://www.ncbi.nlm.nih.gov/refseq/], the Pfam database[https://

pfam.xfam.org/], and the UniProtKB Swiss-Prot database[https://www.uniprot.org/uniprot/?query=reviewed:yes].

Field-specific reporting

Please select the one below that is the best fit for your research. If you are not sure, read the appropriate sections before making your selection.

☐ Life sciences ☐ Behavioural & social sciences ☒ Ecological, evolutionary & environmental sciences

For a reference copy of the document with all sections, see nature.com/documents/nr-reporting-summary-flat.pdf

Ecological, evolutionary & environmental sciences study design

All studies must disclose on these points even when the disclosure is negative.

Study description	This manuscript describes an "Evolve and Re-sequence" study of the copepod Eurytemora affinis complex as well as a population genomic survey of wild populations in the Baltic Sea. Replicate lines (N=10 treatment and N=4 control) were subject to declining salinity for ten generations. Copepods (N=50) were collected for DNA sequencing at generations 0, 6, 10, and 20. Pool-seq was performed on each replicate line and wild population to analyze patterns of genetic diversity and detect signature of natural selection.
Research sample	The Baltic Sea exhibits a natural gradient in salinity. Wild populations were sampled in order to maximize the variation in native salinities sampled. The experimental population was sampled from one of the highest salinity localities in the Baltic Sea in order to maximize the potential evolutionary pressure of low salinity in the laboratory.
Sampling strategy	The number of experimental lines, individuals sampled per lines / population, and sequencing depth were determined based on recommendations from prior studies (e.g. Schlötterer et al. Nat. Rev. Gen. 2014, Kofler & Schlötterer Mol. Biol. Evol. 2014). Copepods were collected from beakers using a water dropper. They were then sorted under a dissecting microscope to ensure an equal sex ratio in each pool and to remove egg sacs from gravid females. Copepods were then placed in RNAlater and frozen at -80C prior to DNA extraction.
Data collection	DNA sequencing data were collected by the University of Chicago Genomics Facility using the Nextera DNA library preparation kit (Illumina, Inc., San Diego, CA, USA). Libraries were sequenced on four lanes of Illumina Hi-Seq 4000 and one lane of Illumina NovaSeq 6000 generating an average of approximately 117 million paired-end (100 bp) reads per pool. Salinity measurements from each sampling site were collected using a handheld refractometer by DBS..
Timing and spatial scale	The E. affinis copepods used in the laboratory natural selection experiment were collected from Kiel Canal in Kiel, Germany (Lat = 54° 19' 59.88"N, Long = 10° 9' 0") in 2017 (approximately 1000 copepods) and on May 30, 2018 (85 gravid females and 40 juveniles). Samples from the laboratory experiment were collected on 7/9/18, 11/22/18, 1/28/19, and 8/12/29. The time points were chosen so that samples were collected at approximately generations 0, 6, 10, and 20 in accordance with our experimental design. The field-collected E. affinis copepods were collected from the Baltic Sea across a salinity gradient (Supplementary Data 3) and preserved in 2018 and 2019. Sequence data were collected from 11/12/18 to 1/28/2020.
Data exclusions	No data were excluded from this analysis.
Reproducibility	All of the scripts needed to reproduce these results are provided in a publicly accessibly Github repository.
Randomization	Laboratory animals were split randomly in bulk from one large population into 14 equally sized populations using a turkey baster. Wild animals were allocated into groups based upon sampling location.
Blinding	Blinding was not relevant to this study, as the relevant data (SNP frequencies) were collected via sequencing machine.

Did the study involve field work? ☒ Yes ☐ No

Field work, collection and transport

Field conditions	Field conditions were typical of the locations and time of year. Average water-column temperature at the time of collection ranged from 11.7 to 17.6 degrees Celsius. Average water column salinity at the time of collection ranged from 2.5 to 6.1 PSU.
Location	Sampling locations and information are presented in Supplementary Data 3.
Access & import/export	The subjects of this study were highly abundant, non-regulated aquatic invertebrates and we did not require permits for collection.
Disturbance	Collections were made using plankton tows, a non-invasive / non-disturbance causing collection method.

Reporting for specific materials, systems and methods

We require information from authors about some types of materials, experimental systems and methods used in many studies. Here, indicate whether each material, system or method listed is relevant to your study. If you are not sure if a list item applies to your research, read the appropriate section before selecting a response.

Materials & experimental systems

n/a	Involved in the study
☒	☐ Antibodies
☒	☐ Eukaryotic cell lines
☒	☐ Palaeontology and archaeology
☐	☒ Animals and other organisms
☒	☐ Human research participants
☒	☐ Clinical data
☒	☐ Dual use research of concern

Methods

n/a	Involved in the study
☒	☐ ChIP-seq
☒	☐ Flow cytometry
☒	☐ MRI-based neuroimaging

Animals and other organisms

Policy information about studies involving animals; ARRIVE guidelines recommended for reporting animal research

Laboratory animals	The E. affinis copepods used in the laboratory natural selection experiment were collected from Kiel Canal in Kiel, Germany (Lat = 54° 19' 59.88"N, Long = 10° 9' 0") in 2017 (approximately 1000 copepods) and on May 30, 2018 (85 gravid females and 40 juveniles).
Wild animals	Copepods were collected using plankton tows. Plankton was brought back to the lab and sorted. Eurytemora affinis complex were pooled and subjected to DNA extraction. Other plankton properly disposed of.
Field-collected samples	Control lines (N=4) were maintained for the duration of the experiment in 15 PSU water made with Instant Ocean, along with Primaxin (20 mg/L) to avoid bacterial infection. The control lines were fed the alga Rhodomonas salina every three-four days with water changed weekly. Treatment lines were fed a 50:50 mixture of R. salina and R. minuta at 5 PSU and only R. minuta at 1 PSU and below.
Ethics oversight	No ethics approval was required because this study used highly abundant, non-regulated invertebrate specimens.

Note that full information on the approval of the study protocol must also be provided in the manuscript.
